# Targeting of Histone Demethylases KDM5A and KDM6B Inhibits the Proliferation of Temozolomide-Resistant Glioblastoma Cells

**DOI:** 10.3390/cancers11060878

**Published:** 2019-06-24

**Authors:** Massimo Romani, Antonio Daga, Alessandra Forlani, Maria Pia Pistillo, Barbara Banelli

**Affiliations:** 1 Laboratory of Tumor Epigenetics, IRCCS Ospedale Policlinico San Martino, 16132 Genova, Italy; alessandra.forlani@hsanmartino.it (A.F.); Mariapia.pistillo@hsanmartino.it (M.P.P.); banellib.epigenetics@gmail.com (B.B.); 2 Laboratory of Cellular Oncology, IRCCS Ospedale Policlinico San Martino, 16132 Genova, Italy; antonio.daga@hsanmartino.it

**Keywords:** glioblastoma, epigenetics, histone methylation, KDM5A, KDM6B, JIB04, GSKJ4, temozolomide, drug resistance

## Abstract

Lysine histone demethylases (KDMs) are considered potential therapeutic targets in several tumors, including glioblastoma (GB). In particular, KDM5A is involved in the acquisition of temozolomide (TMZ) resistance in adult GB cells and UDX/KDM6B regulates H3K27 methylation, which is involved in the pediatric diffuse intrinsic pontine glioma (DIPG). Synthetic inhibitors of KDM5A (JIB 04 and CPI-455) efficiently block the proliferation of native and TMZ-resistant cells and the KDM6B inhibitor GSK J4 improves survival in a model of DIPG. The aim of our work was to determine if GSK J4 could be effective against GB cells that have acquired TMZ resistance and if it could synergize with TMZ or JIB 04 to increase the clinical utility of these molecules. Standard functional and pharmacological analytical procedures were utilized to determine the efficacy of the molecules under study when used alone or in combination against native GB cells and in a model of drug resistance. The results of this study indicated that although GSK J4 is active against native and TMZ-resistant cells, it does so at a lower efficacy than JIB 04. Drug combination studies revealed that GSK J4, differently from JIB 04, does not synergize with TMZ. Interestingly, GSK J4 and JIB 04 strongly synergize and are a potent combination against TMZ-resistant cells. Further studies in animal models will be necessary to determine if this combination of molecules might foster the development of novel therapeutic approaches for glioblastoma.

## 1. Introduction

Histone methylation is a post-synthetic modification of selected lysine of histones H3 and H4 that was discovered along with histone acetylation in the last century [1] and, in conjunction with other histone modifications and DNA methylation, participate in chromatin remodeling [2]. Histone lysine methylation is associated with transcriptionally active or inactive chromatin regions depending on the residue that is modified, and the effect of this modification on transcription is finely tuned by the extent of methylation (mono-, di-, or trimethylation) and from the synergic or antagonist interaction with other modifications [2,3]. In particular, H3 lysine methylation at K4, K36, and K39 marks active chromatin regions, and H3 lysine methylation at K9 and K27 marks inactive chromatin regions [3]. Differently from other histone modifications, lysine methylation does not change the net charge of the histone tail; consequently, the interaction with the DNA was long considered to be an irreversible modification. Only recently, with the discovery of the amine oxidase LSD1 or, according to the current nomenclature, KDM1 [4], it became clear that histone lysine methylation is fully reversible, and we now know that this modification is regulated by more than 30 different enzymes with distinct specificities [5,6].

Being an integral part of the complex histone code, it is conceivable that histone lysine methylation is involved in a multiplicity of physiological mechanisms and pathological conditions like neurological and neurodegenerative disorders [7,8], cardiovascular [9] autoimmune and allergic diseases [10,11], and notably cancer [5,12,13]. Importantly, histone methylation, along with other epigenetic modifications, is deeply involved in the mechanisms of drug resistance, and hence in one of the leading causes of failure of cancer therapy [5,13,14,15,16,17].

Glioblastoma (GB) is a rare tumor (Orphanet 360) that is responsible for 4% of all tumor deaths and is thus one of the deadliest human tumors [18]. Notably, the treatment of glioblastoma has remained essentially unchanged since the introduction, in 2005, of temozolomide (TMZ) in the standard clinical practice [19]. Moreover, the molecularly targeted therapies did not meet the expectations promised by the results obtained in other tumors [20,21]. Intrinsic resistance to radio and chemotherapy, the incomplete surgical resection, the presence of the blood-brain barrier that severely limits the transport of many drugs that are very active on non-brain tumors and, most importantly, the rapid acquisition of chemoresistance are responsible for the treatment failure and for the short survival of glioblastoma patients [22,23,24]. In glioblastoma, epigenetic factors are important drivers of tumor development and response to therapy [25,26,27,28], and epigenetic alterations and epigenetic modifier molecules are being actively exploited as potential targets or effectors of therapy, respectively [25,27,29,30,31,32]. In particular, the H3K4 and H3K9 me1/2 demethylase KDM1 [33,34] and the H3K4 me2/3 demethylase KDM5A have been recognized as potential therapeutic targets and KDM5A was linked to TMZ resistance in GB cells [35]. Importantly, inhibitors of KDM1 and KDM5A enzyme activity display strong antitumor effects against native (WT) and TMZ-resistant (TMZ R) GB cells [33,36] and other tumors [37,38,39,40]. In particular, we showed that the multi-KDM inhibitor JIB 04 [37], which is primarily active against KDM5A, strongly inhibits the growth and activates the autophagic and apoptotic pathways of stem-enriched primary GB cultures that have become resistant to TMZ after treatment with the drug. Furthermore, JIB 04 inhibits the clonogenicity of native and TMZ-resistant GB cells, synergizes with TMZ in killing GB cells, reaches clinically relevant concentrations in the brain and in pilot studies has shown promising activity against GB cells orthotopically xenografted in immunosuppressed mice [36].

The histone variant H3.3 (*H3F3A*) is involved in chromatin remodeling and active transcription [41] and, in pediatric glioma, H3.3 mutations at lysine 27 (K27M) and glycine 34 (G34R/V) occur in approximately 80% of the cases [42] and lead to H3K27 hypomethylation, redistribution of the activation mark H3K26, generalized DNA hypomethylation, and aberrant activation of genes including *MYCN*, which is a driver of gliomagenesis [43,44,45]. Methylation of H3K27 is tightly regulated by the PRC2-EZH2 methylases and by the UTX (KDM6A) and KDM6B demethylases. In an experimental model of diffuse intrinsic pontine glioma (DIPG), an invariably lethal childhood glioma [46], it has been shown that treatment with GSK J4, a potent and selective inhibitor of KDM6A/B, reverts the biological effect of K27M mutation and results in the dramatic improvement of survival in DIPG xenografted mice [45,47]. In a recent study conducted on GB established cell lines, GSK J4 reduced cell proliferation and activated the apoptotic pathway [48], however, the performance of this molecule on freshly derived patient cells and on TMZ-resistant GB cells were not tested. The aims of the present work were to investigate the activity of GSK J4 on native and TMZ-resistant primary and GB established cell lines and to determine if TMZ and GSK J4 or GSK J4 and JIB 04 could synergize in an attempt to develop a cytotoxic/epigenetic model of therapy that is effective against GB cells that have acquired resistance to TMZ.

## 2. Results

### 2.1. Expression of KDM6B in GB Cells

**Figure 1:**
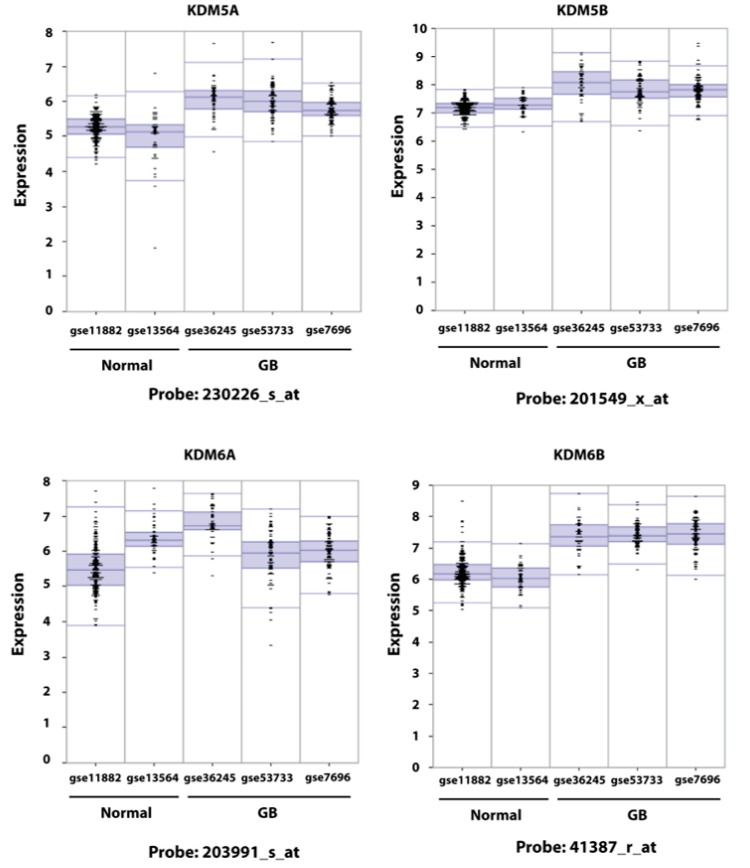
Expression of KDM5A/5B and KDM6A/6B in two normal brain and three glioblastoma datasets. Expression data were from the Affymetrix chip U133 and were analyzed utilizing the R2. package (see Materials and Methods). Four to five probes interrogated each gene. This figure presents the result of the probe indicated below each plot; full data with probe names and *p*-values are reported in Supplementary Table S1.

We have previously shown that, in primary GB, five KDM genes (KDM1A, 4A, 4B, 5A, and 5B) are overexpressed compared to normal brain tissue and that the acquisition of TMZ resistance is accompanied by the further increased expression of KDM5A and, to a lesser extent, of KDM1A, KDM4A, and KDM5B [35]. In that study, KDM6B, a potential therapeutic target of pediatric DIPG, was not examined. To determine if the expression of KDM6A and B was increased in GB, we have investigated in silico the expression of KDM6A and 6B in parallel with that of KDM5A and 5B in three GB datasets (gse7696; gse53733; gse36245) [49,50,51] and in two normal brain datasets (gse13564; gse11882). The datasets utilized for the in silico analysis included a total of 200 samples from GB patients and 216 samples from normal brain tissue. Expression data were obtained utilizing the u133p2 Affymetrix gene-chip; KDM5A, 5B, and 6B were interrogated by five probes and KDM6A by four probes. As shown in Figure 1 and in Supplementary Table S1, this new analysis fully confirmed our earlier report indicating that KDM5A is overexpressed in GB compared to normal brain. Similarly, results indicated that KDM5B was overexpressed, although full concordance across the three GB datasets was observed only for two probes. Importantly, in the two normal brain datasets the expression of these two genes was largely concordant. The pattern of expression of KDM6A and KDM6B was more complex. Although KDM6A expression in normal brain was concordant for three probes out of four, in GB concordant and significant overexpression of this gene was found only for probe 203991_s_a and only when the comparison was made against gse11882. The other comparisons were either not significant or showed decreased expression in tumors. The expression of KDM6B in the two normal brain datasets was significantly different in four out of five probes suggesting that the expression of this gene is highly heterogenous. To exclude the possibility that the different expression of KDM6B could depend on the brain area where the sample was taken from, we interrogated the gse11882 dataset where the samples can be subdivided according to their area of origin (entorhinal cortex, hippocampus, postcentral gyrus, and superior frontal gyrus) and we did not find significant differences of KDM6B expression among them (Supplementary Figure S1). The comparison between tumor and normal datasets revealed a similar complexity, and, overall, only one probe (41387_r_at) showed a strong significant overexpression across all datasets. Nevertheless, the pattern of expression of KDM6B in GB datasets gse53733 and gse7696 and the comparison with the normal brain dataset gse13564 is indicative of the overexpression of this gene in GB, at least in a subset of patients. 

We have previously shown that the expression of KDM1A, 4A, 5A, and 5B is increased upon acquisition of TMZ resistance [35]. We have now extended our previous study to include KDM6B expression in paired native (WT) and Temozolomide-resistant (TMZ R) derivatives of the established cell lines U251 and DBTRG and of the stem-enriched patients’ derived GBM3 cells. U251 and GBM3 TMZ-resistant cells have been previously described [35,36]. A novel TMZ-resistant cell was established from DBTRG cells utilizing the protocol already described [35].

The expression analysis reported in Supplementary Figure S2 shows that KDM6B expression is significantly enhanced in the TMZ derivatives of DBTRG and GBM3 (more than 2-fold increase) but is diminished in U251 TMZ R, suggesting that changes of KDM6B expression are not a constant feature of cells that have acquired TMZ resistance.

### 2.2. GSK J4 Inhibits Proliferation, Activates Apoptosis, and Blocks Cell Cycle Progression in Native and TMZ-Resistant Glioblastoma Cells

GSK J4 is a selective inhibitor of the KDM6 family [52] that was successfully tested in a DIPG model and has shown cell proliferation inhibitory activity on established GB cell lines and promising activities against tumors of different lineages [45,47,48,53,54]. We have now tested the activity of GSK J4 in our model of inducible TMZ resistance to determine if this molecule could be effective against GB cells that acquired resistance to the temozolomide. 

**Figure 2:**
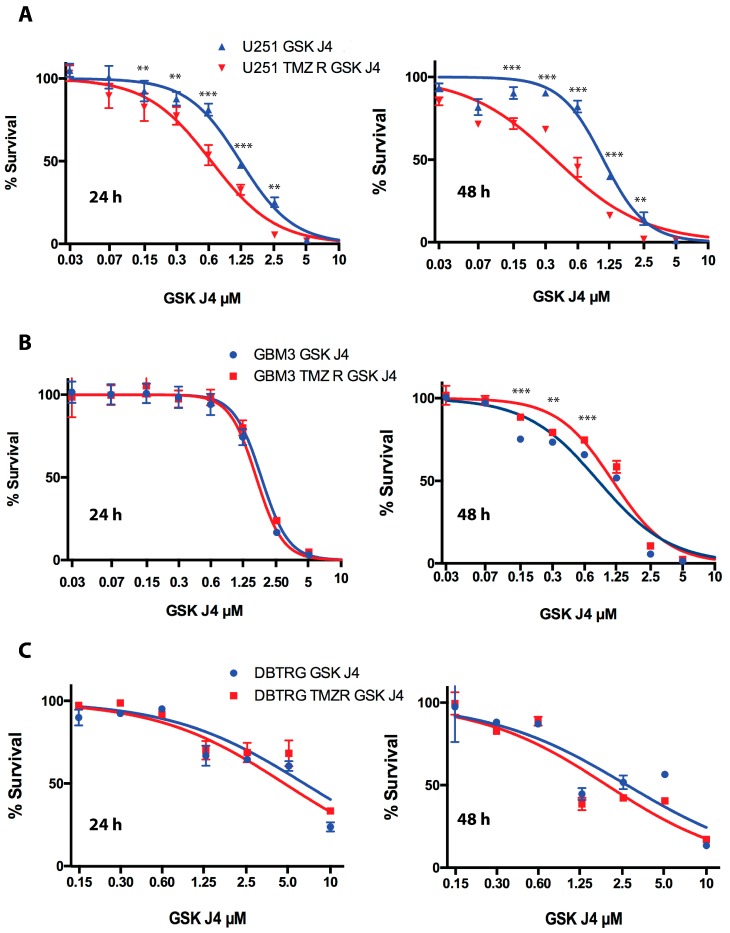
Effect of GSK J4 on cell proliferation. Native and temozolomide (TMZ) resistant U251 (**A**), GBM3 (**B**), and DBTRG (**C**) glioblastoma cells were analyzed in parallel by MTS after 24 and 48 h of treatment with GSK J4 at different concentrations. Statistical analysis was performed by ANOVA, applying Bonferroni post-hoc correction. *p*-values: ** = 0.01; *** = 0.001; no asterisk indicates that the difference was not significant.

The cell proliferation analysis conducted by MTS and reported in Figure 2 shows that TMZ-resistant U251 cells are significantly more sensitive to GSK J4 than their native counterpart in a dose- and time-dependent mode, with IC_50_ of 0.64 and 0.37 µM after 24 and 48 h of treatment versus 1.19 and 1.05 µM of native U251 cells at the same time points (Figure 2A). On the contrary, the sensitivity of GBM 3 to GSK J4 was essentially identical after 24 h (1.65 and 1.79 µM for WT and TMZ cells, respectively) or significantly in favor of WT cells after 48 h (0.81 and 1.1 µM) (Figure 2B). Finally, the sensitivity of DBTRG to GSK J4 did not show significant differences in WT and TMZ derivatives at each drug dosage and time point (IC_50_ 4.6 and 2.6 µM for WT cells vs. 5.1 and 1.9 µM for TMZ R cells at 24 and 48 h, respectively) (Figure 2C).

**Figure 3:**
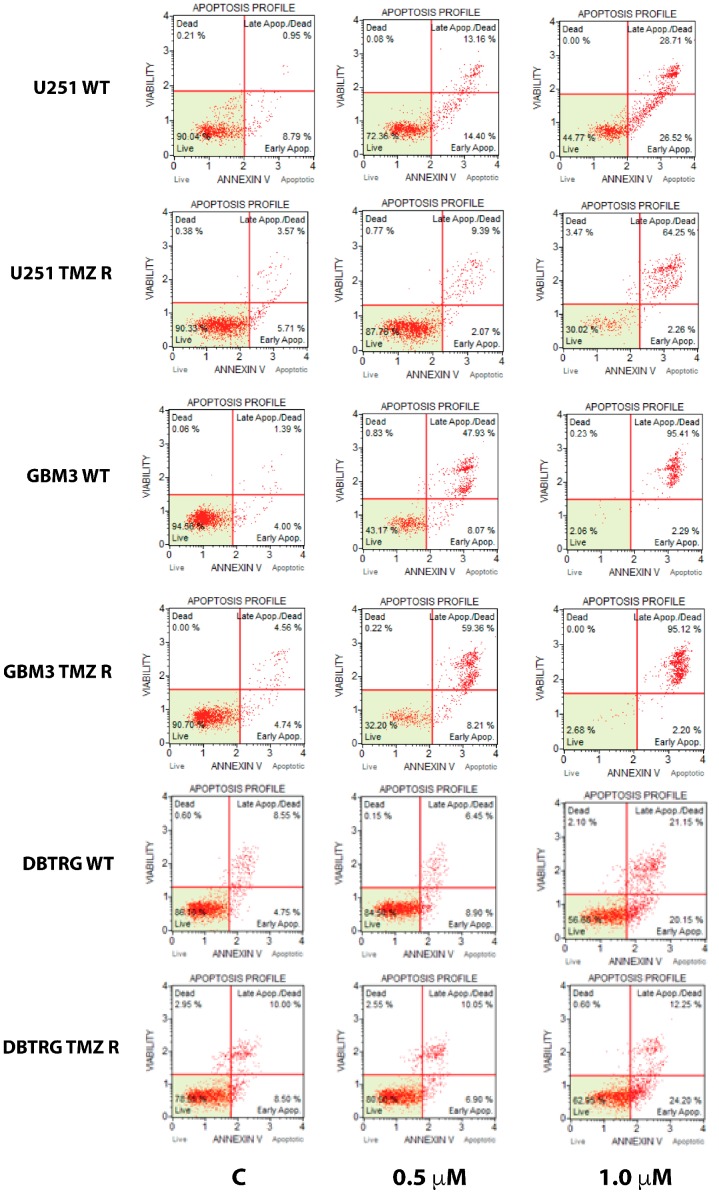
Induction of apoptosis by GSK J4 in native and TMZ-resistant glioblastoma (GB) cells. Representative scatter plots of annexin V staining at two molecule concentrations (0.5 and 1.0 μM) and untreated control (C).

GSK J4 is a potent activator of the apoptotic pathway and, as shown in Figure 3 and in Supplementary Figure S3, the extent of apoptosis induced by this molecule is essentially identical in native and TMZ R cells, and no significant differences were detected by ANOVA after post-hoc analysis.

**Figure 4:**
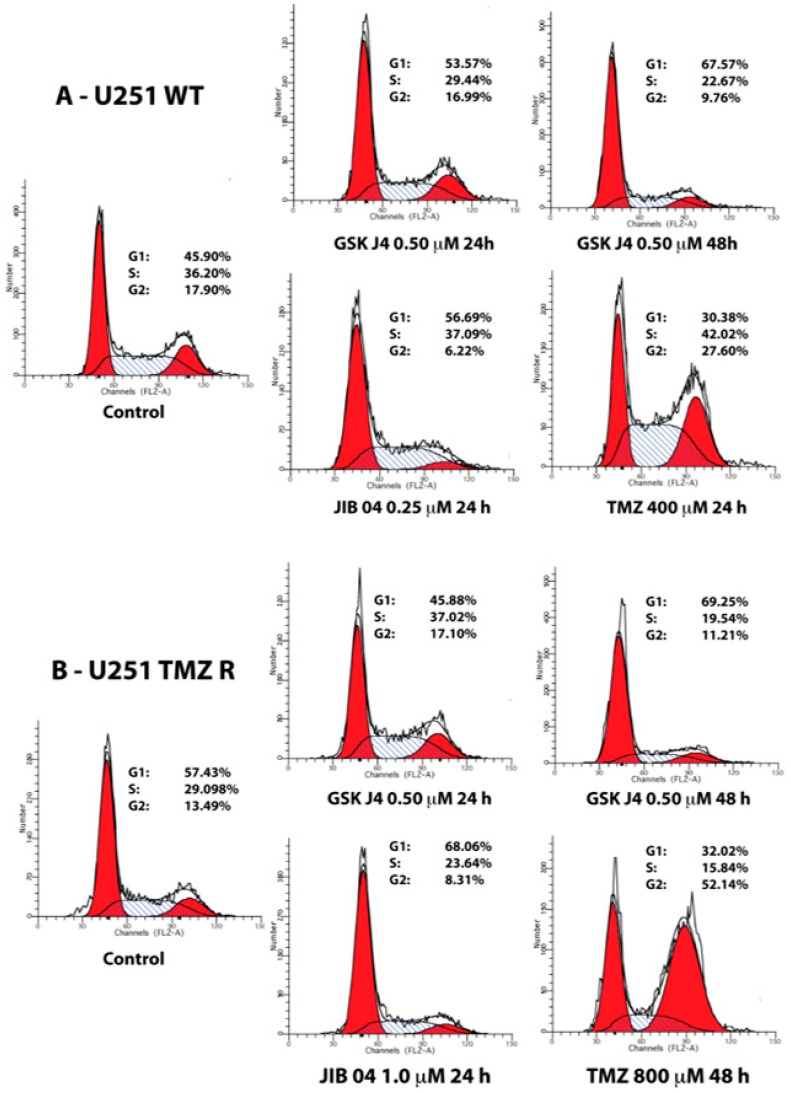
Cell cycle analysis of GB cells treated with TMZ, JIB 04, and GSK J4. The cell cycle distribution of native and TMZ-resistant U251 cells was analyzed by flow cytometry after 24 and 48 h of treatment with the indicated drugs at different concentrations. Representative histogram plots are shown.

Temozolomide blocks cell cycle progression in G2 and JIB 04 in G1, and these two molecules likely synergize because JIB 04 intercepts the cells that escape the G2 checkpoint and enter G1 [35,36]. In the present work, we have essentially confirmed those findings, showing that JIB 04 treatment prevents the progression of the cells into G2 and that TMZ-treated cells accumulate in G2 (Figure 4 and Supplementary Figure S4). Similarly to JIB 04, GSK J4 also strongly reduced the cells’ entry into G2, and cells remained mostly in G1 and S, with the noticeable exception of GBM3 TMZ R cells, where the cells that remained alive during treatment did not show cell cycle alterations and might have represented a subset of cells naturally resistant to GSK J4 (Supplementary Figure S4B).

Overall, these results indicate that GSK J4 inhibits glioblastoma cell growth and activates the apoptotic pathway of native and TMZ-resistant cells. However, no clear-cut differences in sensitivity to this molecule were seen between native and TMZ-resistant cells.

### 2.3. GSK J4 Reduces Clonogenicity in Native and TMZ-Resistant Glioblastoma Cells

We have previously shown that the blockade of the H3K4me3 activator signal through KDM5A inhibition by JIB 04 occurs very rapidly and does not require the constant presence of the molecule. Indeed, the exposure of the cells to JIB 04 for 1 h dramatically diminished the clonogenic property of the cells, and no difference between native and TMZ R cells could be observed [36]. To evaluate the clonogenic properties of GB cells after exposure to GSK J4, we performed a clonogenic assay on native and TMZ R cells utilizing in parallel GSK J4 and JIB 04.

**Figure 5:**
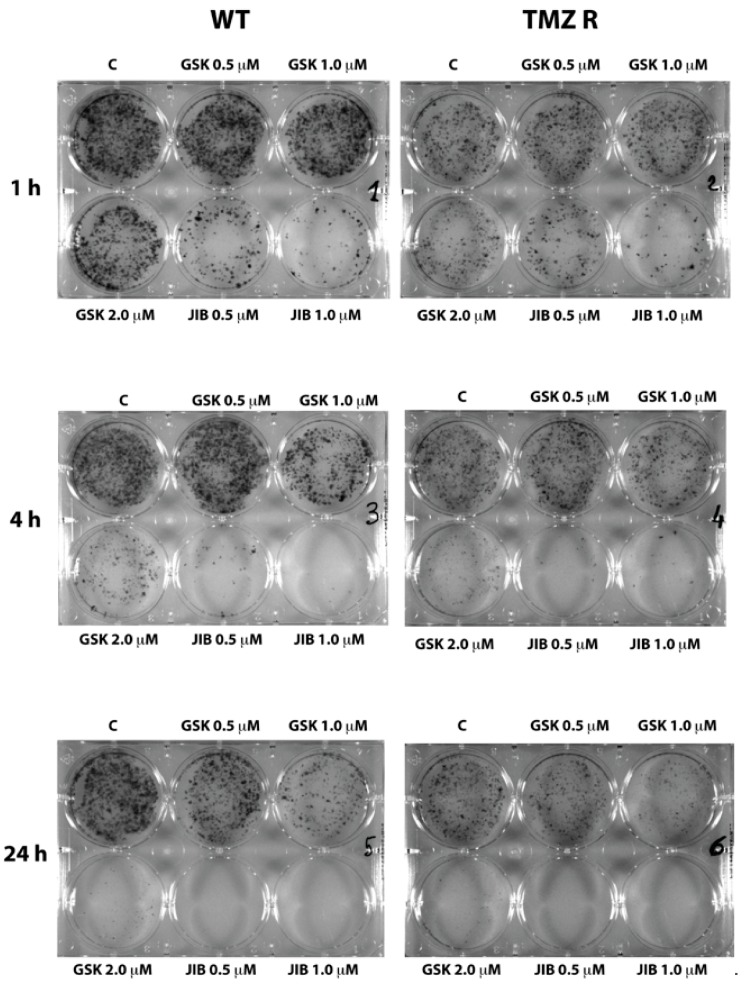
Inhibition of clonogenic activity of GSK J4 and JIB 04 on GBM3 cells. Native and TMZ-resistant GBM3 cells were incubated with different concentrations of KDMi for 1, 4, and 24 h and plated. After 7–10 days, the cells were fixed on plate and stained. The stronger potency of JIB 04 compared to GSK J4 was evident even after 1 h of treatment.

Figure 5 reports the results obtained with WT and TMZ R GBM3 cells and shows that, similarly to JIB 04, GSK J4 is also capable of inhibiting the clonogenicity of GB cells. However, this inhibition occurs at higher concentrations and longer exposure times than JIB 04. Indeed, after 1 h, only marginal inhibition can be detected in WT and TMZ R cells preincubated with GSK J4 at 2 µM, whereas a very strong effect can be seen in cells treated with 0.5 and 1.0 µM JIB 04 for the same time. Only at 4 and 24 h of treatment, at the highest concentration, the effect of GSK J4 became evident; for comparison purposes, we estimated that the effect observed from treating the cells for 24 h with 2 µM GSK J4 was comparable to that observed after 4 h incubation with 0.5 µM JIB 04 before plating.

### 2.4. Cooperation between TMZ, GSK J4, and JIB 04 in Killing Glioblastoma Cells

Cancer chemotherapy is generally carried out by utilizing combinations of drugs with the aim of obtaining a synergic effect to increase the potency of the treatment and to prevent the development of drug-resistant cell clones. Drug combinations could also result in a less potent additive effect or, worse, in antagonism that diminishes the activity of the drugs. All these effects can be mathematically described and quantified utilizing the unifying theorem of Chou-Talalay [55] that numerically express drug interactions in terms of Combination Index (C.I.), where a C.I. = 1 (or log(C.I.) = 0) indicates an additive effect and a C.I. <1 or >1 (or log(C.I.) <0 or >0) indicates synergy or antagonism, respectively.

Because of the rapid acquisition of drug resistance, GB would likely benefit from combined therapies but, until now, no drug combination has been demonstrated to improve survival in GB patients. To explore the possibility of combining a cytotoxic and an epigenetic drug, we have previously shown that, in vitro, the combination of TMZ and JIB 04 exerts synergic effects against WT and TMZ R GB cells [36].

To expand our previous findings, we have utilized the Chou-Talalay experimental design to test the effect of GSK J4 combined with TMZ or JIB 04 on native and TMZ-resistant GB cells. The response to the combined treatment with TMZ and GSK J4 in native and TMZ R cells was variable. The results of the combination experiments reported in Supplementary Figure S5 showed modest synergy at several drug combinations in WT GBM3 cells (log (C.I.): −0.65/0.55), but in most of the cases the effect of the two drugs was additive or antagonist. 

**Figure 6:**
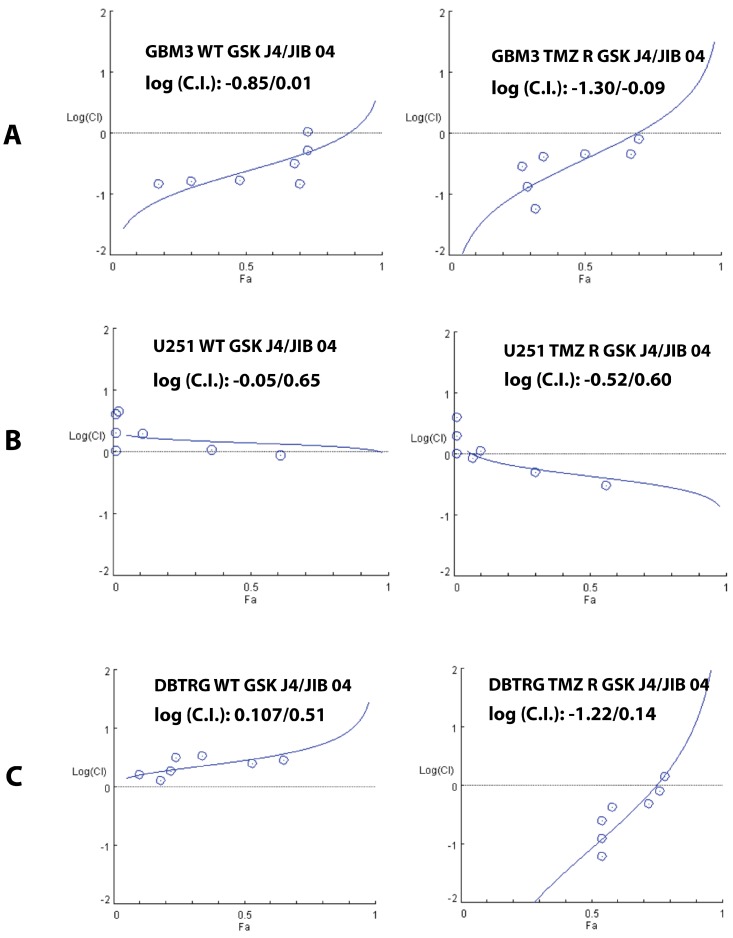
Synergy between GSK J4 and JIB 04 in GBM3, U251, and DBTRG native and TMZ resistant cells. The activity of the molecules was measured by MTS. The results are expressed as Plot of the log(C.I.) index vs. the effect (Fa), where log(C.I.) ≅ 0 indicates an additive effect, whereas log(C.I.): >0 or <0 indicates antagonism or synergy, respectively. (**A**) Combination experiments where GSK J4 and JIB 04 were added simultaneously and the cells were grown for 48 h. Both combinations were strongly synergic for native and TMZ-resistant cells, but this combination was more active against TMZ-resistant cells compared to native cells (log(C.I.): −0.85/0.01 for WT and −1.30/−0.09 for TMZ R cells). (**B**) Combination experiments conducted as in (A) with U251 cells. On WT cells, this combination was largely additive or antagonist at most concentrations (log(C.I.): −0.05/0.65). On the contrary, the effect on TMZ R cells was synergic at several concentrations (log(C.I.): −0.52/0.60). (**C**) Combination experiments conducted as in (A) with DBTRG cells. In this case, the combination was antagonist in WT cells (log(C.I.): 0.107/0.51) and strongly synergic in the TMZ-resistant derivative (log(C.I.): −1.22/0.14).

We next tested GSK J4 and JIB 04 and found that, overall, this combination was synergic in both native and TMZ R GBM3 cells but was more effective against TMZ-resistant than native cells (log(C.I.): −0.85/0.01 for WT and −1.30/−0.09 for TMZ R cells) (Figure 6A). In native U251 and DBTRG cells, this combination was mostly additive or antagonist (log (C.I.): −0.05/0.65 for native U251 and log (C.I.): 0.107/0.51 for DBTRG), on the contrary, the effect of GSK J4 combined with JIB 04 also showed synergy against the temozolomide-resistant derivatives of U251 and DBTRG (Figure 6B,C).

We then confirmed the synergy between GSK J4 and JIB 04 in clonogenic assays where the cells were treated with the two molecules, alone at 0.2 and 0.4 µM (24 h), and in combination at 0.2 µM for each molecule. Drug concentration and treatment time (1 h) were chosen in preliminary experiments to minimize the effect when the molecules were utilized as single agents. As shown in Supplementary Figure S6, the clonogenic properties of the cells were essentially unmodified by the treatment with GSK J4 or JIB 04, but were dramatically reduced when the two molecules were combined.

In conclusion, these experiments suggest that GSK J4 and JIB 04 synergize in cells that have acquired resistance to TMZ. The molecular mechanisms that could explain the synergic effect of GSK J4 and JIB 04 against TMZ R cells up to now are unknown and, in principle, the possibility that these molecules could hit secondary and yet undetermined targets cannot be ruled out.

## 3. Discussion

In spite of intensive research, glioblastoma remains one of the deadliest human tumors, and essentially no progress has been made since the discovery of the clinical utility of adjuvant chemotherapy with the alkylating agent temozolomide, which is able to improve patients’ survival for a few months [19]. The rapid acquisition of radio and chemoresistance, coupled with the impossibility of performing a radical surgery and with the persistence of tumor cells in niches protected by the blood-brain barrier [56], have so far prevented the discovery of more efficient therapies for this tumor. In recent years, epigenetic and immunological therapies received considerable attention because of their potential in overcoming the pitfalls of chemo- and molecularly targeted therapies (for recent reviews, see [32,57]).

We have recently shown that the H3K4me3 demethylase KDM5A endogenous or exogenous overexpression is associated with transient and reversible resistance to TMZ, and that the inactivation of KDM5A by shRNA restores TMZ sensitivity in drug-resistant cells [35]. Furthermore, we have also shown that the polyspecific KDM inhibitor JIB 04 (primarily active against KDM5A [37]) and the specific KDM5 inhibitor CPI-455 [39] preferentially inhibit the proliferation of TMZ-resistant GB cells [36]. Based on our in vitro model, we proposed that JIB 04 can intercept and kill the cells that escape the G2 cell cycle block exerted by TMZ. Intuitively, adding a third molecule acting on a different pathway could increase the chances of establishing a successful therapeutic protocol. We thus selected GSK J4, a selective H3K27m3 UDX/KDM6B inhibitor, that passes the blood-brain barrier and showed promising activity against pediatric DIPG [45,47].

We first performed an extensive in silico analysis to determine the expression pattern of KDM5A and KDM6B in several GB datasets. The results of this first series of experiments confirmed the overexpression of KDM5A in GB and showed a remarkable concordance between the three datasets examined and the multiple probes that interrogated the assay platforms. On the contrary, the pattern of expression of KDM6B in GB patients was highly variable, suggesting that the tumors are highly heterogeneous; moreover, we could not find an unequivocal relation between TMZ resistance and KDM6B expression, suggesting that this gene does not have a central role in the development of TMZ resistance. Nevertheless, KDM6B appears as a potential target for experimental therapeutics in GB, since inhibiting KDM6B (and KDM6A) activity with GSK J4 blocked cell proliferation, induced apoptosis in native and TMZ R cells, and prevented cell cycle progression and entrance in G2. In this respect, the effects of GSK J4 are similar to those of JIB 04.

To explore the possibility that GSK J4 could have, in principle, a clinical utility in adult GB, we evaluated the antitumor effect of GSK J4 and JIB 04 in combination with TMZ and found that GSK J4 only marginally synergizes with TMZ. Interestingly, the combination of JIB 04 and GSK J4 seems to be the most effective against native and TMZ-resistant cells and appears to be a good candidate for in vivo studies in animal models either as a first line treatment in combination with TMZ or as a second line treatment after the standard TMZ treatment.

## 4. Materials and Methods

### 4.1. In Silico and Statistical Analysis

GB and normal brain datasets were interrogated utilizing the R2 Genomic Analysis and Visualization Platform (https://hgserver1.amc.nl). Statistical significance was determined by analysis of variance (ANOVA) utilizing the statistical utility built-in in the R2 package. The other statistical analyses were performed by ANOVA applying Bonferroni post-hoc correction for multiple comparisons, utilizing the Graph Pad Prism software (GraphPad Software, San Diego, CA, USA).

### 4.2. Cell Lines, Cell Cultures, and Drugs

The human GB cell line U251, the stem-enriched GBM3 cultures [58,59], and the generation of their TMZ-resistant derivatives has been already described [35,36]. A new TMZ-resistant cell derivative was generated from DBRG cells by utilizing the same treatment protocol described for the other cells. Cell lines were obtained from the Biological Bank and Cell Factory (www.iclc.it) of IRCCS Ospedale Policlinico San Martino (Genova, Italy) and were grown in DMEM supplemented with 10% FBS and 2 mM L-glutamine. The authenticity of the cells was certified by the Biological Bank utilizing eight highly polymorphic short tandem repeats loci plus amelogenin (Cell IDTM, Promega, Milano, Italy).

Temozolomide was acquired from Sigma-Aldrich (Milano, Italy), JIB 04 was acquired from Tocris (Bristol, UK) or Selleckchem (Munich, Germany), and GSK was acquired J4 from ABCAM (Cambridge, UK).

### 4.3. Cellular Analyses

Cell proliferation and viability were measured by colorimetric MTS (One Solution Cell Proliferation Assay, Promega, Milano, Italy). Apoptosis, measured as annexin V staining, was determined utilizing a MUSE cell analyzer (Millipore-Merck, Vimodrone, Milano, Italy) utilizing the dedicated kits. Clonogenic assays were performed as described by Franken et al. [60].

### 4.4. Cell Cycle Analysis

Cells were washed in PBS, fixed with ice-cold 95% ethanol at 4 °C for 1 h, and resuspended in staining solution (PBS, 20 mg/mL RNase A, 50 mg/mL propidium iodide, 0.5% Triton X-100, all from Sigma-Aldrich). DNA content was quantified by FACScalibur (BD Bioscience, Milano, Italy) equipped with BD CellQuest Pro software. Cell-cycle profile was determined by analysis of Listmode data using ModFit LT software (Verity Software House, https://www.vsh.com/). At least 10,000 events were collected, gating single nuclei and excluding aggregates

### 4.5. Quantitative Real-Time Reverse Transcription-PCR

Real-Time PCR analysis was performed as previously described [35]. KDM6B primer sequences were derived from Ramadoss et al. [61]. Relative quantification of KDM6B was obtained using the comparative Ct method. *ATP5b*, *SDHA1*, and *CYC1* were selected as reference genes using GeNorm (PrimerDesign Ltd., Southampton, UK). The fold induction was expressed relative to a calibrator sample using the 2^−(ΔΔCt±SD)^ method.

### 4.6. Drug Interaction

Drug interaction experiments were conducted by MTS by testing TMZ in combination with either JIB 04 or GSK J4 and JIB 04 in combination with GSK J4, at various concentrations in a constant ratio according to the protocol of Chou and Talalay [55], utilizing the Compu-Syn software (www.combosyn.com).

## 5. Conclusions

We have demonstrated that the KDM6 inhibitor GSK J4 inhibits cell growth, clonogenicity, and cell cycle progression and activates apoptosis in native and TMZ-resistant glioblastoma cells. Importantly this molecule synergizes with the multi-KDM inhibitor JIB 04 and is a potent inhibitor of GB cells that have acquired resistance to TMZ. We thus hypothesize that GSK J4 could be useful as a second-line drug in conjunction with JIB 04 when resistance to TMZ begins to appear. Appropriate testing in animal models will be necessary to develop treatment schedules to balance the strong antitumor effects of these molecules with their toxicity. Nevertheless, we believe that targeting KDMs offers new potential weapons against GB, a tumor for which few and inefficient therapeutic options exist.

## Supplementary Materials

The following are available online at https://www.mdpi.com/2072-6694/11/6/878/s1, Figure S1: In silico expression analysis of KDM6B in four normal brain areas, Figure S2: KDM6B expression in native and TMZ resistant GB cells determined by qPCR analysis, Figure S3: Induction of apoptosis by GSK J4 in GB cells, Figure S4: Cell cycle analysis of GB cells treated with TMZ, JIB 04, and GSK J4, Figure S5: Combination experiments between TMZ and GSK J4, Figure S6: Synergic effect between GSK J4 and JIB 04 detected by clonogenic assay, Table S1: Statistical evaluation of the expression differences between glioblastoma and normal brain datasets for each Affymetrix probe.
