# Supplementary material for: Targeting of Histone Demethylases KDM5A and KDM6B Inhibits the Proliferation of Temozolomide-Resistant Glioblastoma Cells
Source: Cancers (Basel). 2019 Jun 24;11(6):878. doi: 10.3390/cancers11060878 (PMC6627323; doi:10.3390/cancers11060878)
Supplement: Supplementary file 1 [file cancers-11-00878-s001.pdf]

Supplementary Materials

Targeting of Histone Demethylases KDM5A and KDM6B Inhibits the Proliferation of Temozolomide-Resistant Glioblastoma Cells

Massimo Romani, Antonio Daga, Alessandra Forlani, Maria Pia Pistillo and Barbara Banelli

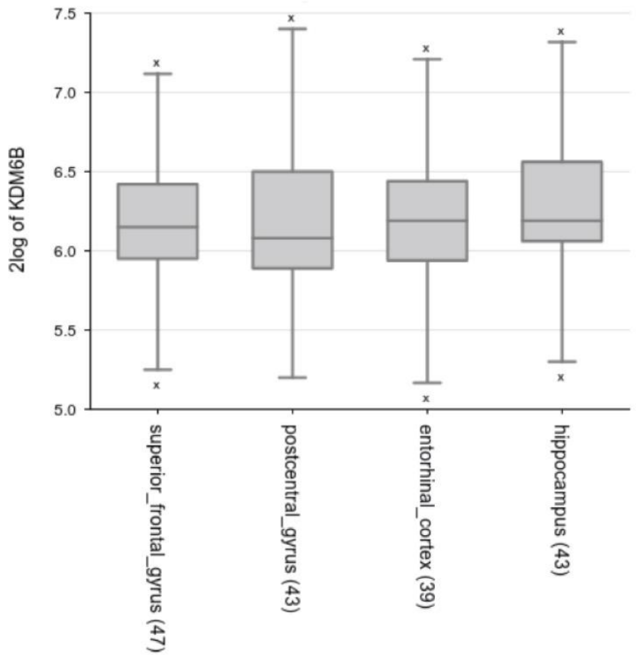

Figure S1. In silico expression analysis of KDM6B in four normal brain areas. Expression data were from the Affymetrix chip and were analyzed utilizing the R2 package (see Materials and Methods). In this figure is represented the result of probe 41387_r_at.

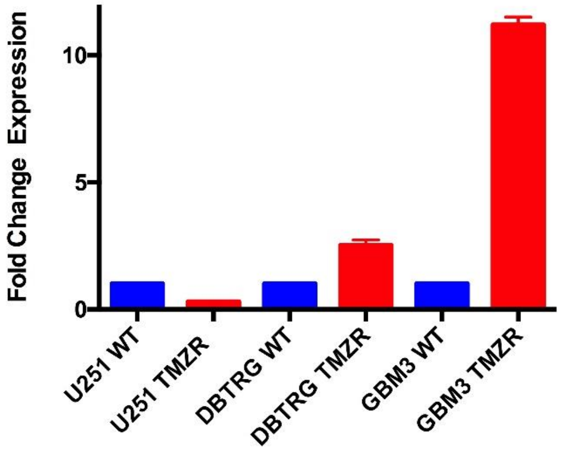

Figure S2. KDM6B expression in native and TMZ resistant GB cells determined by qPCR analysis. Cells were exposed to GSK J4 (IC50 for 24 h), mRNA extracted from WT and TMZ resistant cells was analyzed in parallel in two independent experiments; each sample was analyzed in triplicate. Results are expressed as fold change of expression respect to the untreated control whose expression was set to 1.

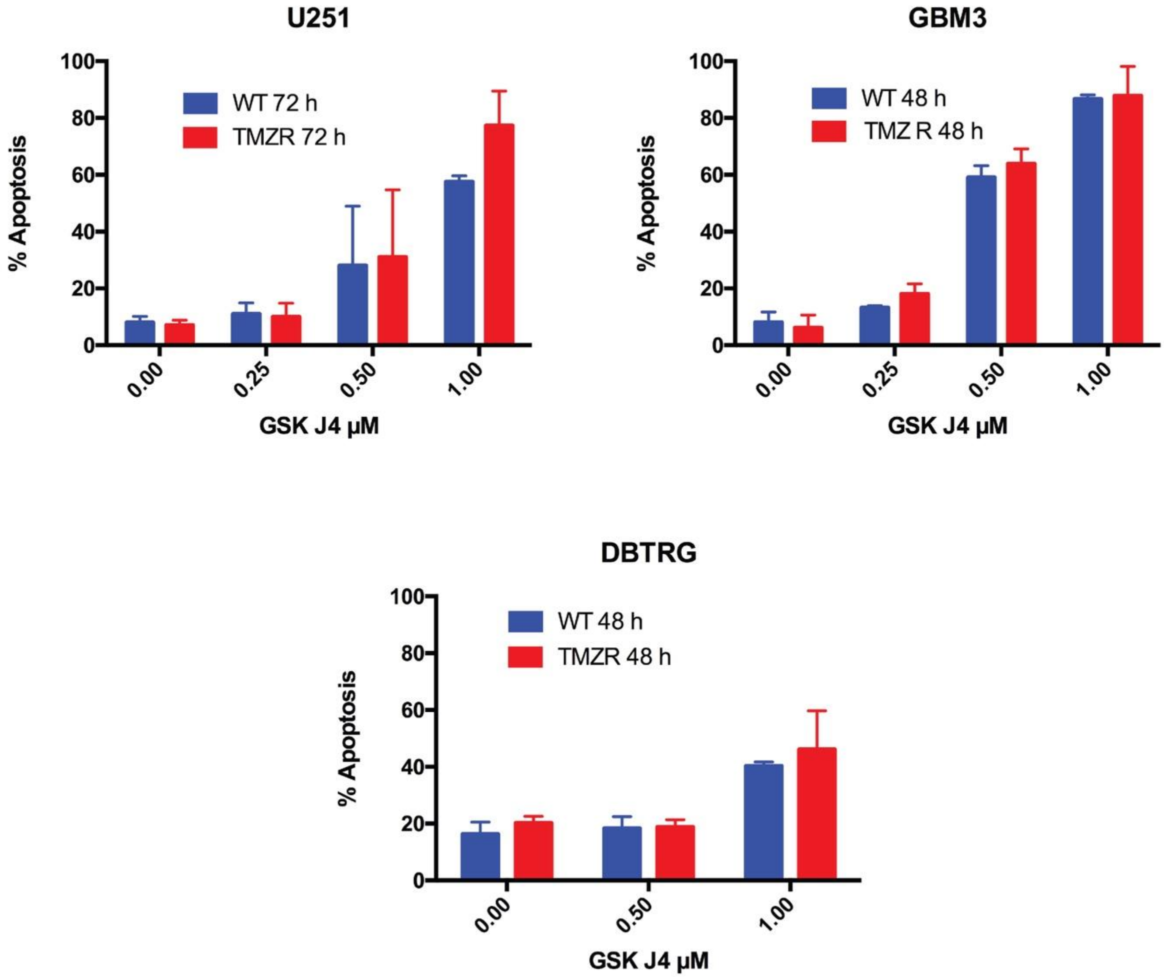

Figure S3. Induction of apoptosis by GSK J4 in GB cells. The cells were incubated with various concentrations of GSK J4 for the time indicated in the panels and apoptosis was measured by Annexin V staining as indicated in Materials and Methods. Panels report the mean total apoptosis (early + late) and each value represents the mean of triplicate (DBTRG) or quadruplicate (GBM3 and U251) independent experiments. No significant differences between WT and TMZR cells were detected by ANOVA and post-hoc analysis (Bonferroni).

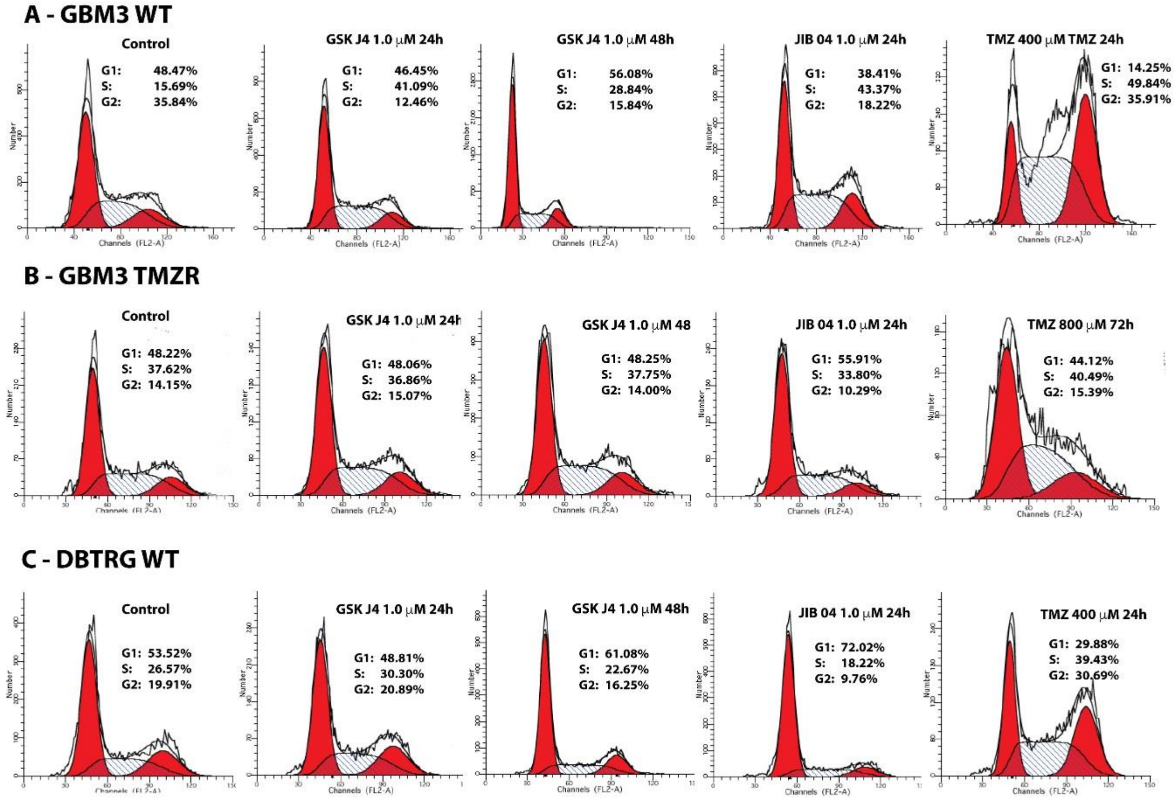

Figure S4. Cell cycle analysis of GB cells treated with TMZ, JIB 04, and GSK J4. (**A**) GSK J4 or JIB 04 treatment on GBM3 WT cells induced a transient reduction of G2 after 24 h that exited in a G1 accumulation at 48h. TMZ increased the percentage of S and G2 phases just after 24 h of treatment. (**B**) GBM3 TMZR cells showed a reduced sensitivity to all the drugs used but modified the cell cycle distribution in response to high concentrations of TMZ only after 72 h of treatment. (**C**) DBTRG cells responded to all drugs as GBM3 WT.

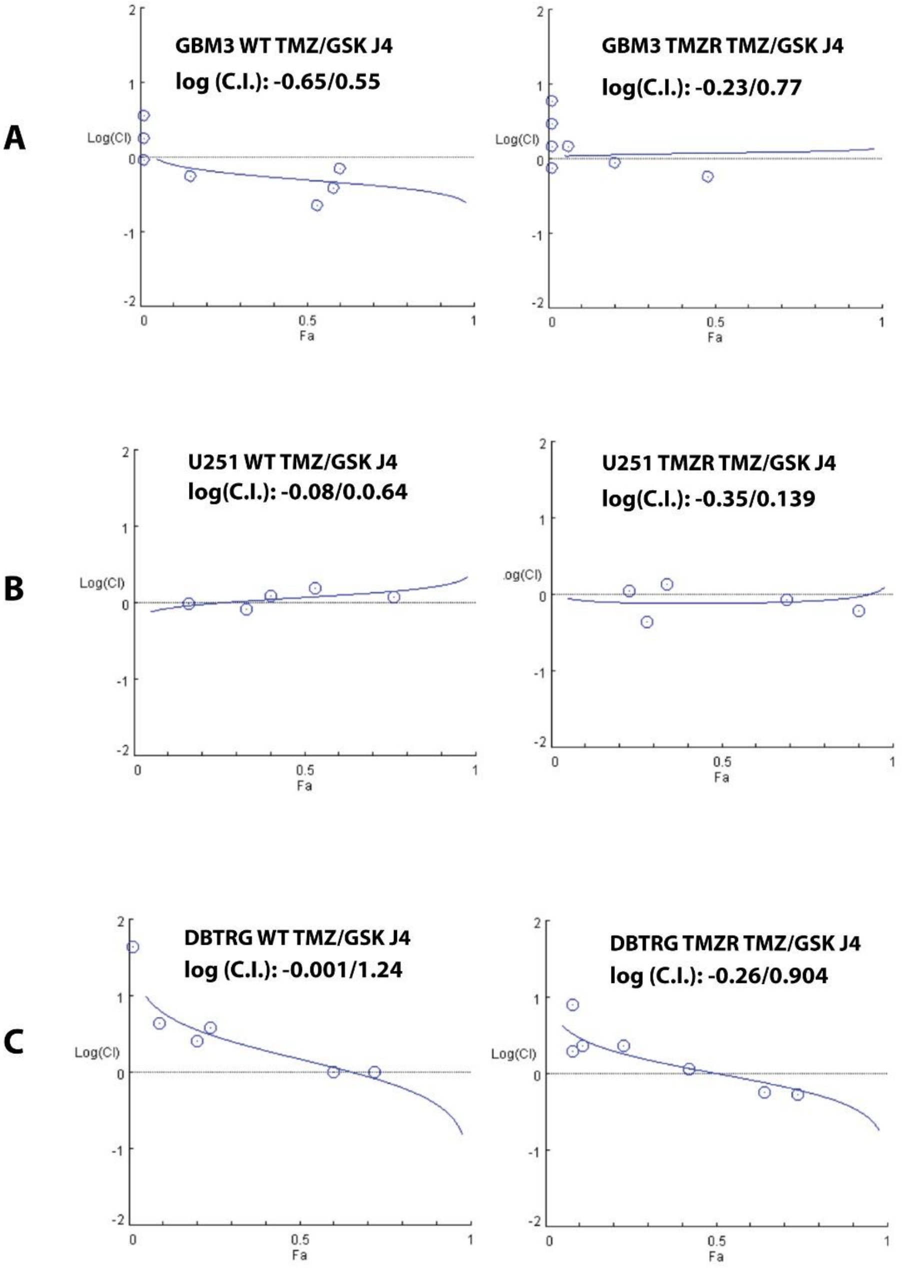

Figure S5. Combination experiments between TMZ and GSK J4. The experiments were conducted as described in Figure 5 and in Materials and Methods and show that in most of the cases the log(C.I.) values are indicative of additive or antagonist effects, Only in GBM3 WT cells several combination points suggest an additive effect between the two molecules. All other combinations are close to log(C.I.) = 0 and only sparse points are indicative of synergy between these molecules.

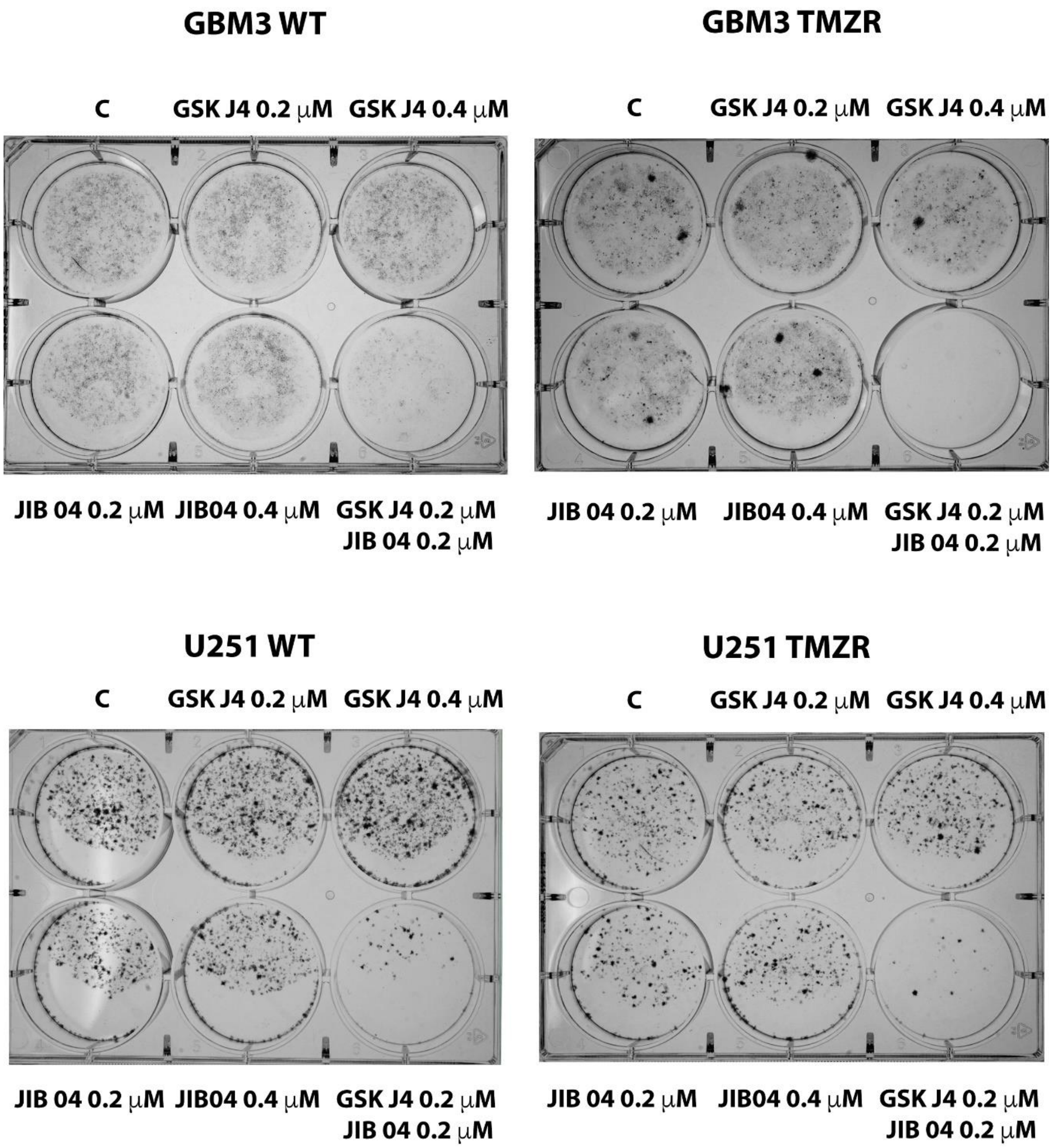

Figure S6. Synergic effect between GSK J4 and JIB 04 detected by clonogenic assay. The activity of the combination GSK J4/JIB 04 was determined in clonogenic assays in GBM3 and U251 cells incubated for 1hour with the concentration of drugs indicated in the figure or untreated (C). The dramatic reduction of the number of cell clones in the well containing GSK J4 and JIB 04 compared to those containing only one of the molecules essentially confirms the results described in Figure 5 obtained with a different methodology.

Table S1. Statistical evaluation of the expression differences between glioblastoma and normal brain datasets for each Affymetrix probe.

**KDM5A**				
**Probe**	**Normal Datasets**	**Glioblastoma vs. Normal Datasets**		
	gse13564 vs. gse11882	gse36245 vs. gse11882	gse53733 vs. gse11882	gse7696 vs. gse11882
202040_s_at	0.17	2.4e-22	1.6e-54	4.6e-07
215698_at	0.11	1.2e-13	1.4e-59	2.7e-05
226367_at	0.41	7.5e-21	6.9e-24	2.4e-03
226371_at	1.0e-09	2.4e-37	5.7e-53	1.5e-20
230226_s_at	2.4e-04	9.5e-26	1.1e-30	4.5e-24
		gse36245 vs. gse13564	gse53733 vs. gse1356	gse7696 vs. gse1356
202040_s_at		9.0e-20	4.5e-36	2.1e-09
215698_at		1.0e-12	5.4e-36	1.9e-06
226367_at		8.3e-10	4.9e-10	0.21
226371_at		2.4e-18	4.9e-26	7.5e-17
230226_s_at		5.5e-12	1.6e14	1.4e-13
**KDM5B**				
**Probe**	**Normal Datasets**	**Glioblastoma Datasets**		
	gse11882 vs. gse13564	gse36245 vs. gse11882	gse53733 vs. gse11882	gse7696 vs. gse11882
201547_at	0.02	5.4e-03	1.1e-07	1.7e-03
201548_s_at	6.0e-04	3.1e-13	2.1e-04	0.34
201549_x_at	4.6e-03	3.6e-32	4.2e-30	2.9e-34
211202_s_at	0.01	5.1e-25	1.5e-28	1.8e-40
235285_at	2-0e-04	1.9-e06	2.6e-05	5.8e-10
		gse36245 vs. gse13564	gse53733 vs. gse13564	gse7696 vs. gse13564
201547_at		9.2e-24	8.3e-07	0.94
201548_s_at		3.2e-08	8.9e-05	0.01
201549_x_at		6.6e-10	1.2e-08	9.2e-10
211202_s_at		8.0e-08	8.1e-09	9.7e-13
235285_at		6.7e-06	2.6e-06	0.74
**KDM6A**				
**Probe**	**Normal Datasets**	**Glioblastoma Datasets**		
	gse11882 vs. gse13564	gse36245 vs. gse11882	gse53733 vs. gse11882	gse7696 vs. gse11882
203990_s_at	0.03	8.3e-17	2.7e-07	2.4e-29
203991_s_at	7.2e-14	7.0e-26	4.8e-04	1.9e-08

203992_s_at	0.22	0.16	0.18	0.97
238220_at	0.26	4.7e-03	0.34	1.1e-03
		gse36245 vs. gse13564	gse53733 vs. gse13564	gse7696 vs. gse13564
203990_s_at		1.1e-08	0.02	6.5e-11
203991_s_at		7.0e-05	6.2e-05	4.9e-05
203992_s_at		0.05	0.97	0.24
238220_at		0.21	0.08	0.20
**KDM6B**				
**Probe**	**Normal Datasets**	**Glioblastoma Datasets**		
**KDM6B**	gse11882 vs. gse13564	gse36245 vs. gse11882	gse53733 vs. gse11882	gse7696 vs. gse11882
41386_i_at	2.0e-39	8.6e-43	6.5e-11	9.2e-10
41387_r_at	0.2	5.6e-30	4.9e-47	9.3e-47
213146_at	2.6e-10	1.5e-35	0.45	2.7e-40
1556066_at	5.0e-27	6.4e-20	7.0e-03	0.32
1556067_a_at	7.6e-0.9	1.4e-04	2.0e-05	6.3e-05
		gse36245 vs. gse13564	gse53733 vs. gse13564	gse7696 vs. gse13564
41386_i_at		0.04	5.6e-16	1.2e-65
41387_r_at		1.3e-21	9.6e-36	8.9e-31
213146_at		9.5e-08	4.9e-08	8.4e-07
1556066_at		0.6	1.2e-17	1.2e-15
1556067_a_at		0.13	9.9e-14	0.02

At first the two normal brain datasets were compared, then each tumor dataset was compared separately against the two normal brain datasets and the *p*-value level for each probe is recorded. Black numbers indicate overexpression compared to the normal tissue, red numbers indicate lower expression compared to normal tissue.

© 2019 by the authors. Licensee MDPI, Basel, Switzerland. This article is an open access article distributed under the terms and conditions of the Creative Commons Attribution (CC BY) license (http://creativecommons.org/licenses/by/4.0/).
